# Genetic trajectory and clonal evolution of multiple primary lung cancer with lymph node metastasis

**DOI:** 10.1038/s41417-022-00572-0

**Published:** 2023-01-19

**Authors:** He Tian, Yalong Wang, Zhenlin Yang, Ping Chen, Jiachen Xu, Yanhua Tian, Tao Fan, Chu Xiao, Guangyu Bai, Lin Li, Bo Zheng, Chunxiang Li, Jie He

**Affiliations:** 1 Department of Thoracic Surgery, National Cancer Center/National Clinical Research Center for Cancer/Cancer Hospital, Chinese Academy of Medical Sciences, Peking Union Medical College, Beijing, 100021 China; 2 Department of Anesthesiology, National Cancer Center/National Clinical Research Center for Cancer/Cancer Hospital, Chinese Academy of Medical Sciences, Peking Union Medical College, Beijing, 100021 China; 3 Department of Medical Oncology, Yancheng No. 1 People’s Hospital, Yancheng, Jiangsu 224000 China; 4 Department of Medical Oncology, National Cancer Center/National Clinical Research Center for Cancer/Cancer Hospital, Chinese Academy of Medical Sciences and Peking Union Medical College, Beijing, China; 5 Guangdong Provincial People’s Hospital/Guangdong Provincial Academy of Medical Sciences, Guangdong Provincial Key Lab of Translational Medicine in Lung Cancer, Guangzhou, China; 6 Department of Thoracic Surgery/Head & Neck Medical Oncology, UT MD Anderson Cancer Center, Houston, TX 77030 USA; 7 Department of Pathology, National Cancer Center/National Clinical Research Center for Cancer/Cancer Hospital, Chinese Academy of Medical Sciences, Peking Union Medical College, Beijing, 100021 China

**Keywords:** Non-small-cell lung cancer, Cancer genomics

## Abstract

Multiple primary lung cancer (MPLC) with lymph node metastasis (LNM) is a rare phenomenon of multifocal lung cancer. The genomic landscapes of MPLC and the clonal evolution pattern between primary lung lesions and lymph node metastasis haven’t been fully illustrated. We performed whole-exome sequencing (WES) on 52 FFPE (Formalin-fixed Paraffin-Embedded) samples from 11 patients diagnosed with MPLC with LNM. Genomic profiling and phylogenetic analysis were conducted to infer the evolutional trajectory within each patient. The top 5 most frequently mutated genes in our study were TTN (76.74%), MUC16 (62.79%), MUC19 (55.81%), FRG1 (46.51%), and NBPF20 (46.51%). For most patients in our study, a substantial of genetic alterations were mutually exclusive among the multiple pulmonary tumors of the same patient, suggesting their heterogenous origins. Individually, the genetic profile of lymph node metastatic lesions overlapped with that of multiple lung cancers in different degrees but are more genetically related to specific pulmonary lesions. SETD2 was a potential metastasis biomarker of MPLC. The mean putative neo-antigen number of the primary tumor (646.5) is higher than that of lymph node metastases (300, *p* = 0.2416). Primary lung tumors and lymph node metastases are highly heterogenous in immune repertoires. Our findings portrayed the comprehensive genomic landscape of MPLC with LNM. We characterized the genomic heterogeneity among different tumors. We offered novel clues to the clonal evolution between MPLC and their lymphatic metastases, thus advancing the treatment strategies and preventions of MPLC with LNM.

## Introduction

According to the newest data published in 2022 [1], the lung cancer death rate is 21% worldwide, ranking the first in both genders among all cancer types. Over 350 people die of lung cancer every day. Lung cancer burdens public healthcare annually [2, 3].

In recent decades, multifocal lung cancer has been an increasingly common scenario in clinical practice [4, 5]. It is reported that up to 15% of lung cancer patients developed two or more lesions [6], among which multiple primary lung cancer (MPLC) is very common [7]. MPLC indicates patients who developed two or more pulmonary tumors that were originally independent, and the multiple pulmonary lesions can be synchronous or metachronous [8, 9]. In 2016, the International Association for the Study of Lung Cancer (IALSC) classified multifocal lung cancer into four categories: (1) Second primary cancer, (2) Separate Tumor Nodules (Intrapulmonary metastasis, IPM), (3) Multifocal Lung Adenocarcinoma with Ground Glass/Lepidic (GG/L) Features and (4) Diffuse Pneumonic Type [10], among which second primary cancer and multifocal GG/L are commonly acknowledged as MPLC by clinicians. So far, there are no guidelines or widely accepted criteria in the realm of MPC.

Identifying the “dominant” lung lesions with the most malignant nature is crucial for MPLC patients, many of whom are not qualified for radical surgical resection because of operational contraindication, such as advanced age or prolonged anesthesia risk. Pre-locating the critical tumor before surgery can provide those patients with more treatment options. From the view of evolution, MPLC with lymph node metastasis (LNM) is an ideal material for studying the genetic trajectory of MPLC, thus identifying the “dominant” lung lesions. However, there are several barriers to the study of MPLC with LNM. Firstly, precise discrimination between MPLC and lung cancer intrapulmonary metastasis (IPM) has been a clinical dilemma for decades [6, 11]. Both two diseases behave as multiple tumor sites in the lung, but they are of distinct staging strategies [8], treatment [9–11], and prognosis [8, 12, 13]. Secondly, MPLC with LNM is very rare in clinical practice. Thirdly, the difficulties in sample collection of MPLC with LNM hamper its studies.

What we know about MPLC with LNM is far from enough, though some researchers have paid attention to it. In 2016, Gao et al. focused on an MPLC patient with one lymph node metastasis and applied targeted panel sequencing on him [12]. The lymph node metastasis shared 52 common mutations with one of the pulmonary tumors but had no overlap with other lung lesions within the individual. A similar conclusion was observed in Omada et al.’s research in 2020 [13], in which five MPLC with LNM patients were included.

Our study performed WES on tumor samples from 11 patients diagnosed as MPLC with LNM. We are the first to characterize the clonal evolution pattern of MPLC with lymph node metastasis using comprehensive genetic sequencing. Our findings may help grasp the mysterious nature of MPLC and identify the tumor lesion most likely to metastasize before surgery, thus spurring the introduction of MPLC guidelines.

We present the following article by the MDAR reporting checklist.

## Methods and materials

### Study design and patients

According to the 8th edition of the TNM staging system published in 2017, a total of 1458 patients during 2011 and 2019 who were diagnosed with MPLC receiving no adjuvant treatment before surgery were identified in Cancer Hospital, the Chinese Academy of Medical Sciences, and Peking Union Medical College. Under the following criteria: (1). Positive lymph node metastasis; (2). Complete access to FFPE specimens of pulmonary tumors, lymph node metastasis tumors, and matched normal samples, 11 patients were finally included in our study. Two independent certified pathologists confirmed the diagnosis by HE staining and provided the pathological details of each patient (Table 1). The clinical information was retrieved from the medical record system of our hospital (Table 2). CT (Computerized Tomography) images were obtained from the department of radiology of our hospital, and two experienced radiological specialists annotated the pulmonary tumor lesions independently. The ethics committee of Cancer Hospital, Chinese Academy of Medical Science approved the study, and written informed consent was obtained from each involved patient. The analysis was performed in accordance with the local ethical regulations and the guidelines of the Declaration of Helsinki.

**Table 1:** Tumor Identification, Pathological Subtype, and Sequencing information of 11 patients.

Patient ID	Sample ID	Tumor Location	Tumor Size (mm)	Histology	Pathological Subtype					TNM Stage	WES Depth/X (Mean)
					A%	P%	MP%	S%	L%		
1	N	RLL	—	Normal	—					—	346
	T1	RLL	17*13*13	ADC				100		T2	383
	T2	RLL	10*10*9	ADC	10			90		T1b	306
	LN1	Lower Lobe Parabronchial	—	ADC				100		N1	289
	LN2	Region 7	—	ADC				100		N2	311
	LN3	Upper Lobe Parabronchial	—	ADC				100		N1	334
	LN4	Middle Lobe Parabronchial	—	ADC	10	10		80		N1	327
2	N	RUL	—	Normal	—					—	321
	T1	RUL	12*10*10	ADC		80	20			T1b	313
	T2	RUL	10*10*7	ADC	100					T1a	221
	LN1	Extrapulmonary	—	ADC	100					N1	351
	LN2	Region 4 R and 2 R	—	ADC	30	70				N2	313
	LN3	Region 11	—	ADC	100					N1	NA
3	N	RUL	—	Normal	—						367
	T1	LLL	13*12*14	ADC	20	80				T1b	295
	T2	RUL	24*15*15	ADC			100			T1c	NA
	LN1	LUL Parabronchial	—	ADC			100			N1	261
	LN2	LLL Extrapulmonary	—	ADC			100			N1	183
	LN3	Region 3 A	—	ADC			100			N2	NA
4	N	LUL	—	Normal	—					—	360
	T1	LUL	25*15*10	ADC	30			70		T1c	330
	T2	LUL	22*9*3	ADC	60			40		T1c	331
	T3	LUL	12*10*10	ADC	30			70		T1c	292
	LN1	Region 5 and 6		ADC	20			80		N2	300
5	N	RUL	—	Normal	—					—	NA
	T1	RUL	26*17*10	ADC				100		T2	261
	T2	RUL	30*26*20	ADC				100		T1c	NA
	LN1	Region 10	—	ADC				100		N1	258
	LN2	Region 11	—	ADC				100		N1	269
	LN3	Region 4 R	—	ADC				100		N2	274
6	N	RLL	—	Normal	—					—	358
	T1	LUL	25*22*20	ADC	10	10		70	10	T2	276
	T2	LLL	15*13*5	ADC				70	30	T2	348
	T3	RLL	9*6*5	ADC		80		20		T1a	331
	LN1	Region 5	—	ADC	10	10		80		N2	NA
7	N	RLL	—	Normal	—					—	NA
	T1	RLL	10*5*3	ADC		30	70			T1a	234
	T2	RUL	30*25*30	ADC	40				60	T1c	NA
	LN1	Extrapulmonary	—	ADC	100					N1	435
	LN2	Region 4 R	—	ADC	100					N2	299
8	N	RUL	—	Normal	—					—	299
	T1	RUL	15*13*10	ADC	90	10				T1b	281
	T2	RUL	29*20*18	ADC	30	30		40		T2	306
	LN1	Region 4 R	—	ADC		10		90		N2	365
9	N	RLL	—	Normal	—					—	487
	T1	RLL	65*35*35	ADC				100		T3	328
	T2	RUL	14*12*2	ADC				80	20	T3	363
	LN1	Subcarinal	—	ADC				100		N2	306
10	N	LUL	—	Normal	—					—	392
	T1	LUL	30*30*22	ADC		100				T2	237
	T2	LUL	10*5	ADC	95	5				T1a	NA
	LN1	Parabronchial	—	ADC				100		N1	344
	LN2	Parabronchial	—	ADC	100					N1	270
	LN3	Region 5	—	ADC				100		N2	394
11	N	LUL		Normal	—					—	63
	T1	LUL	32*28*15	ADC		5		95		T2	72
	T2	LUL	17*10*7	ADC		20		80		T1b	69
	LN1	Extrapulmonary	—	ADC				100		N1	92
	LN2	Intrapulmonary	—	ADC			10	90		N2	70
	LN3	Proximal Posterior Basal	—	ADC				100		N1	84
	LN4	Region 10	—	ADC				100		N1	115

Failed, WES was not performed because of failure in cDNA library construction. NA, unknown because of WES failure.

*N* normal sample, *T* tumor sample, *LN* lymph node metastasis, *RUL* right upper lobe, *RLL* right lower lobe, *LUL* left upper lobe, *LLL* left lower lobe, *ADC* adenocarcinoma, *A* acinar, *P* papillary, *MP* micropapillary, *S* solid, *L* lepidic, *H* high, *M* middle, *L* low, *M–L* middle to low, *H–M* high to middle.

**Table 2:** Demographic information and clinical characteristics of 11 patients with MPLC.

Patient ID	Age/Gender	Ethnicity	Smoking, PY	Alcohol, y	Family History	Pattern	Surgery	Comprehensive Classification*	Adjunct Therapy	Patient Status^#^	Recurrence/Distant Metastasis	Follow-up/months
1	42/F	Han Chinese	0	0	Yes	Syn	Thoracotomy	Primary	ALK Targeted therapy	Alive	Recur	108
2	68/F	Han Chinese	0	0	Yes	Syn	VATS	Primary	None	Alive	None	67
3	45/F	Han Chinese	0	0	No	Syn	VATS	Primary	EGFR Targeted therapy	Alive	Bone Metastasis	75
4	56/M	Han Chinese	35	0	No	Syn	VATS	Primary	Chemotherapy	Alive	None	60
5	76/M	Han Chinese	0	20	No	Syn	VATS	Primary	Chemotherapy +EGFR Targeted therapy	NA	NA	NA
6	48/F	Han Chinese	0	0	No	Meta	VATS	Primary	None	Dead	NA	78
7	64/F	Han Chinese	0	0	No	Syn	VATS	Primary	None	Alive	None	85
8	52/M	Han Chinese	0	25	Yes	Syn	VATS	Primary	None	Dead	Recur	28
9	65/M	Han Chinese	45	30	Yes	Syn	Thoracotomy	Primary	None	Dead	Recur	8
10	73/M	Han Chinese	33	0	No	Syn	VATS	Primary	EGFR Targeted therapy+Radiotherapy	Alive	Brain Metastasis	87
11	67/M	Han Chinese	0	0	No	Syn	VATS	Primary	Capecitabine+Icotinib	Alive	None	15

*F* female, *M* male, *PY* packed-years, *y* years, *Syn* synchronous, *Meta* metachronous, *NA* unknown, *Recur* recurrence, *VATS* video-assisted thoracoscopic surgery.

*Patients are classified according to the 8th TNM staging system published in 2017.

^#^Until the last follow-up time.

### WES sequencing

We extracted total DNA from archived FFPE samples using the QIAamp DNA FFPE Tissue Kit (Qiagen, cat. no. 56404). To enhance the tumor purity, two independent pulmonary pathologists marked tumor areas on FFPE slides, and the non-tumor components were scraped off before DNA extraction. DNA from paired normal lung tissue samples was used to eliminate the influence of germline mutation. Isolated genomic DNA quality was verified using three combined methods: (1) DNA degradation, and contamination was monitored on 1% agarose gels. (2) DNA purity was checked using the NanoPhotometer® spectrophotometer (IMPLEN, CA, USA). (3) DNA concentration was measured by Qubit® DNA Assay Kit in Qubit® 2.0 Fluorometer (Invitrogen, USA). WES libraries were prepared using Agilent SureSelect Human All Exon V6 kit (Agilent Technologies, CA, USA). Following the manufacturer’s recommendations, index codes were added to each sample, and the clustering of the index-coded samples was performed on a cBot Cluster Generation System using Hiseq PE Cluster Kit (Illumina). DNA libraries were sequenced on the Illumina Hiseq platform with a paired-end 2 × 150 protocol. The WES was conducted at CapitalBio Technology Inc. Beijing, China, from January 2020.01 to October 2020.

### Data processing and bioinformatics

#### Sequence alignment, and variant calling

We trimmed and filtered raw data using Trimmomatic 0.33 [14]. Paired-end clean reads were aligned to the human reference sequence hg19 using the BWA-MEM algorithm (BWA version 0.7.10-r789) with default parameters [15]. To guarantee meaningful downstream analysis, duplicated sequencing reads were excluded by Picard v.2.13. In contrast, low-confidence reads (reads containing adapter contamination, low-quality nucleotides, and unrecognizable nucleotide (N)) were removed by the criteria of TLOD < 10. All high-confident mutations were then annotated into the MAF format using the tool vcf2maf. We called somatic single-nucleotide variants (SNVs) and small indels using MuTect (version 3.1-0-g72492bb) and Strelka (version 1.0.14) [16, 17]. All mutations in coding regions were manually checked using the Integrative Genomics Viewer (version2.3.34) [18].

#### Definitions of putative driver mutations

We compared all non-silent mutations with lists of lung cancer/pan-cancer potential driver genes in the COSMIC cancer gene census (September 2021). We identified the driver mutations in our data if they are in the lists.

#### Mutation spectra and signature analyses

We extracted each mutation’s 5′ and 3′ sequence context from the hg19 reference genome (BSgenome.Hsapiens.UCSC.hg19). We categorized the SNVs into C > A, C > G, C > T, T > A, T > C, and T > G bins according to the type of substitution and then subcategorized them into 96 sub-bins according to the nucleotides preceding (5′) and succeeding (3′) the mutated base. The deconstructSigs package (v1.8.0) was used to infer the contributions of 30 published signatures from the Catalog of Somatic Mutations in Cancer (COSMIC) (https://cancer.sanger.ac.uk/cosmic/signatures_v2) in each sample [19]. The contribution of each signature for each tumor was statistically quantified.

#### Phylogenetic tree construction and labeling

All nonsynonymous somatic mutations were considered for determining phylogenetic trees. We built trees using binary presence/absence matrices generated from the distribution of variants within different tumors within each patient. R Bioconductor package phangorn [20] was used to perform the parsimony ratchet method, generating unrooted trees. Branch and trunk lengths were proportional to the number of nonsynonymous mutations. To label the clonal phylogenetic trees for each patient, we defined 5 categories of mutations according to the driver genes lists in the COSMIC cancer gene census (September 2021): (1) Tier 1 NSCLC/lung cancer driver genes. (2) Tier 2 NSCLC/lung cancer driver genes. (3) Tier 1 pan-cancer driver genes. (4) Tier 2 pan-cancer driver genes. (5) Genes not on the list.

#### Unsupervised clustering

The hclust function (the agglomeration method is “ward.D2”) in R software (Version 4.0.2) was utilized to perform unsupervised clustering. According to the driver gene list from the Catalog of Somatic Mutations in Cancer (COSMIC) (https://cancer.sanger.ac.uk/cosmic/signatures_v2), a totally of 40 genes were utilized, including 20 genes with the highest mutation frequencies in our data, 10 pan-cancer driver genes with top mutation frequencies except for the top 20 genes, and 10 lung cancer driver genes with top mutation frequencies except for the top 20 genes.

#### Functional enrichment analysis

We applied the 468 genes from the MSK-IMPACT assay to our cohort and conducted GO functional enrichment analysis, and showed the essential genes.

#### Venn diagram and upset graph

Venn diagrams and Upset charts were used to illustrate the mutational overlaps among multiple samples within individuals. For patients with no more than 5 tumor samples, we drew the Venn diagrams using Omicshare (an online tool, https://www.omicshare.com/tools/Home/Soft/venn). For Patient 1 and Patient 11, each of whom has 6 tumor samples, the R software package UpSetR was applied to draw Upset graphs.

#### Putative neoantigens identification and binding affinity prediction

Polysolver algorithm [21] was applied to conduct HLA typing. Non-silent mutations were used to generate a list of mutant peptides of approximately 9–11 amino acids in length, with the mutated residues represented in each position.

NetMHCpan (v3.0) was used to predict the binding affinity of each mutant peptide and its corresponding wild-type peptide to the patient’s germline HLA alleles [22]. Candidate neoantigens were identified with a predicted mutant peptide binding affinity of <500 nmol/L and rank<2.

### Statistical analysis

Statistical analysis was performed using Fisher’s exact test for categorical variables and the Wilcoxon or Mann–Whitney U test for continuous variables. We estimated the mutual exclusivity of mutations by Monte Carlo simulation. We calculated the relevance between constant variables using Pearson’s correlation or Spearman’s correlation. Kaplan–Meier curves and the log-rank tests were used for the survival analysis. Statistical tests were performed in R (v3.2.0) and GraphPad (v8). We regarded a two-tailed *P* value < 0.05 as statistically significant.

## Results

### Patient cohort

**Fig. 1:**
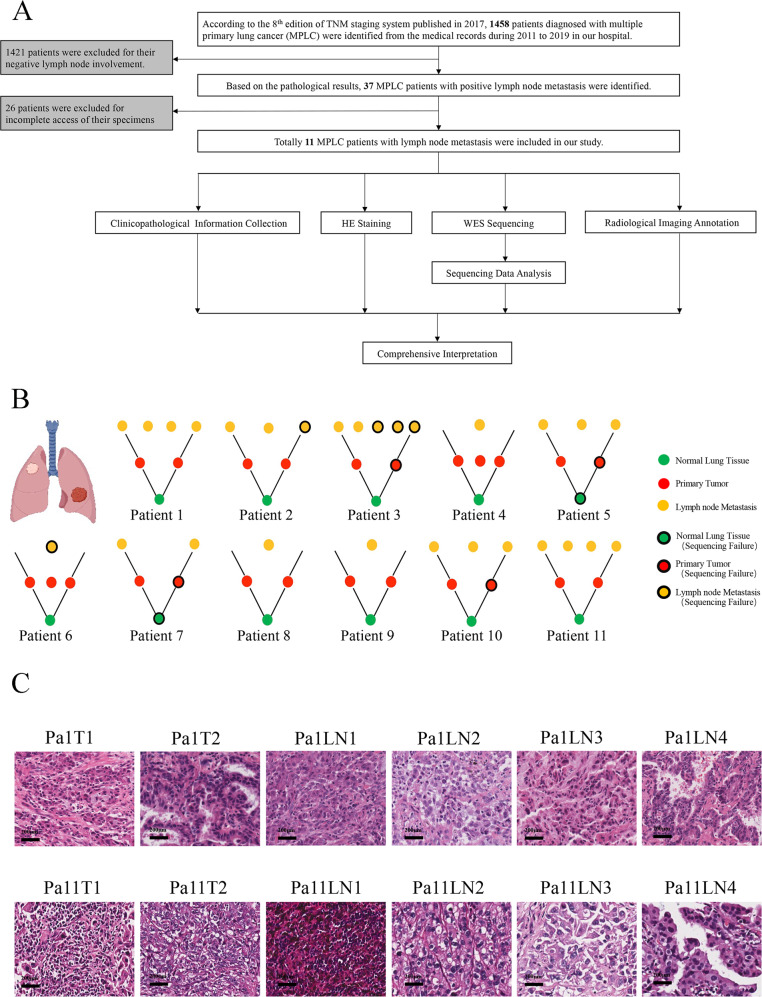
Study design and patient description. **A** The flow chart showed the design and workflow of our research. **B** The cartoons showed the concept of multiple primary lung cancer, the different colors of the two tumors indicated they are genetically independent. Pattern diagrams constituted by dots of different colors showed the tumor architecture within each patient. The green, red, and orange dots represented standard samples, primary lung tumors, and lymph node metastases sites. The black circle indicated that the sample existed but failed to produce valid data in WES. **C** Representative HE staining image of patient 1 and patient 11 (Magnification: 100X. Scale bars: 200 μm).

The workflow was presented in Fig. 1A. Initially, 1458 patients were selected, based on whom 1421 patients were excluded for they lacked lymph node metastasis, and 26 patients were excluded for their FFPE samples were not available. Finally,11 MPLC with LNM patients were recruited.

We explored these patients from clinicopathological information, hematoxylin-eosin (HE) staining, WES sequencing, and radiological images. The ideograph of all 11 patients was displayed in Fig. 1B. Representative HE images of Patients 1 and 11 were shown in Fig. 1C (Supplementary Fig 1).

### Clinicopathological and demographic information

The clinicopathological details and WES depth were in Table 1. We defined the pathological subtype with the highest proportion as the major pathological component. In 72.73% (8/11) patients, the multiple lung tumors were of heterogenous major pathological components, while in Patient1, 5, and 11, all their pulmonary tumors were solid subtypes. In 81.2% (9/11) patients, we captured pathological consistency between primary tumors and metastases, but in Patient7 and 10, their lymph node metastasis sites were heterogenous to primary tumors pathologically. Stage annotations were based on the proposed TNM classification criteria of lung cancers with multiple pulmonary sites by IASLC in 2016 [6]. Most lung tumors were staged as T1 and T2, and most lymph node metastatic lesions were staged as N1 or N2. The average sequencing depth of tumor and normal samples were 275x (range 69x-435x) and 332x (range 63x-487x), respectively. Table 2 exhibits demographic information. According to the proposed differentiation criteria of lung cancer with multiple pulmonary sites of involvement by IASCL in 2016 [10], all patients in our study were MPLC. The average age of our cohort was 59.6 years old (range 42-76). 27.27% (3/11) patients had a smoking history, and 27.27% (3/11) patients were alcohol users. Over one-third of patients (36.36%, 4/11) had a positive family history, especially Patient 9, who had a familial history of both lung and prostate cancer. All patients were synchronous MPLC, except Patient6 (metachronous MPLC). The post-surgery recurrence/metastasis rate was 55.56% (5/9) patients. The average follow-up time of all patients is 61.1 months. In this study, both the primary lung/pulmonary tumor samples and the lymph node metastases tumor samples of each patient are identified as the tumor samples of this patient. We emphasize the different locations of tumor samples when the analysis requires us to separate the tumor samples into primary lung/pulmonary tumor samples and lymph node metastases tumor samples, during which the location-based integration logic is identical for all patients.

### Genomic alterations

**Fig. 2:**
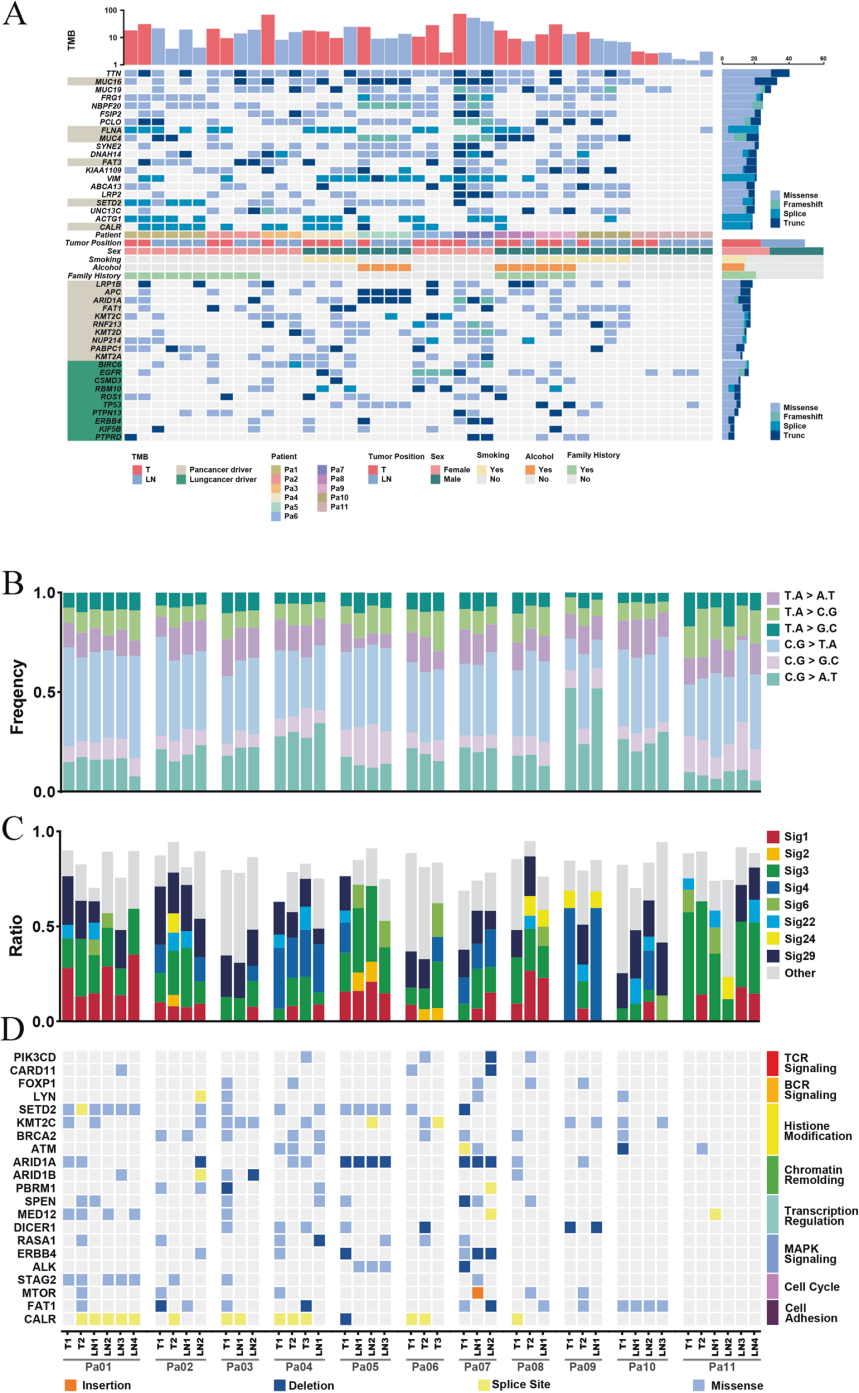
Mutational landscape of MPLC with lymph node metastasis. **A** Significantly mutated genes (SMGs). Upper: Top 20 genes with the highest mutation frequency. Middle: Demographic and clinical information of the 11 patients. Bottom: Highly mutated pan-cancer driver genes (light brown) and lung cancer driver genes (green), ranked according to their frequency. Driver gene identification is based on the COSMIC Cancer Gene Census (Sep. 2021, https://cancer.sanger.ac.uk/census). **B** Six-type mutation spectra of all samples. **C** Signature contributions of all samples based on the COSMIC database. **D** Putative driver genes (based on MSK-IMPACT 468 gene panel) with somatic mutations in all 11 patients were classified according to the functional categories.

We successfully conducted WES on 52 DNA samples from 11 patients to assess genomic alterations globally. Sequencing quality information of all the samples was in Supplementary Data 1, and the list of somatic nonsynonymous mutations was in Supplementary Data 2. The alteration spectrum is Fig. 2A (Supplementary Data 3). According to the driver gene list from COSMIC Cancer Gene Census (Sep. 2021, https://cancer.sanger.ac.uk/census, Supplementary Data 4), we marked the pan-cancer driver genes (light brown) and lung cancer/NSCLC driver genes (green). The mean tumor mutation burden (TMB) for pulmonary tumors and lymph node metastases was 12.78 and 8.94 mutations per megabase, respectively.

Among the top 20 genes with the highest alteration rates, 30% (6/20) were pan-cancer driver genes. TTN (Titin, 76.74%), MUC16 (Mucin16, 62.79%), and MUC19 (Mucin19, 55.81%) were the 3 genes with the highest alteration rates in our cohort. Pan-cancer driver genes with high alteration rates include FLNA (Filamin A, 41.86%), MUC4 (Mucin 4, 41.86%), and FAT3 (FAT Atypical Cadherin 3, 39.53%). Lung cancer/NSCLC driver genes with high alteration rates include BIRC6 (Baculoviral IAP Repeat Containing 6, 30.23%), EGFR (Epidermal Growth Factor Receptor, 30.23%), and TP53 (Tumor Protein P53, 20.93%). SETD2 (SET Domain Containing 2, Histone Lysine Methyltransferase) is the histone H3 lysine 36 histone (H3K36) methyltransferase [23] and has been reported in various solid tumors and blood malignancies [24–26]. In our cohort, SETD2 alternated in all 6 tumor samples (2 primary tumors and 4 lymph node metastasis) of the Patient1, including 5 splice site mutations and 1 missense mutation. Other genes alternating in both primary tumors and lymph node metastasis tumors within individuals include CALR (Calreticulin), FRG1(FSHD Region Gene 1), and ACTG1 (Actin Gamma 1).

The single-nucleotide variations (SNVs) displayed considerable variations across and within patients, indicating intratumor heterogeneity (Fig. 2B, Supplementary Data 5). For Patient 4, 9, and 10 with smoking history, most of their sequenced samples displayed a preponderance of C > A transitions, which is associated with tobacco exposure [27]. In the Patient 9, the SNV pattern of Patient 9LN1 is highly similar to that of Patient 9T1 rather than Patient 9T2, implying the metastasis dominance of Patient 9T1. The contributions of various known signatures to each sample are demonstrated in Fig. 2C (Supplementary Data 6). Signature 4 was prevalent in Patient4, 9, and 10, consistent with the fact that they were smokers. However, signature 4 was also observed in non-smoker patients, and signature 29 (representing tobacco chewing) was prevalent in a large proportion of samples, suggesting that tobacco exposure may play a critical role in the pathogenesis of MPLC with LNM. Signature 3 was identified in 93.20% (40/43) of tumor samples, indicating that DNA mismatch repair was highly involved in the etiology of MPLC with LNM. The signatures echoes between primary tumors and lymph node metastasis tumors offer clues for identifying the dominant primary tumor. For example, in the Patient 1, signature 22 only appeared in T1 and LN1, suggesting their unique connection. In the Patient 9, the signatures contribution of LN1 was almost identical to that of T1, implying that T1 might be the source of LN1. Based on the MSK-IMPACT 468 panel genes (Supplementary Data 7), we acquired 315 genes from our data and conducted functional enrichment analysis (Fig. 2D, Supplementary Data 8). Several signaling pathways were involved, including histone modification, chromatin remolding, MAPK, and cell cycle.

Overall, the mutation spectrum of MPLC with lymph node metastasis was distinct from that of typical lung adenocarcinoma, indicating the unique genomic profile of this disease. Meanwhile, primary tumor and lymph node metastasis echo in both SNVs and COSMIC signature levels, showing the potentiality of clarifying metastasis trajectory and identifying the dominant primary tumor.

### Genomic heterogeneity

**Fig. 3:**
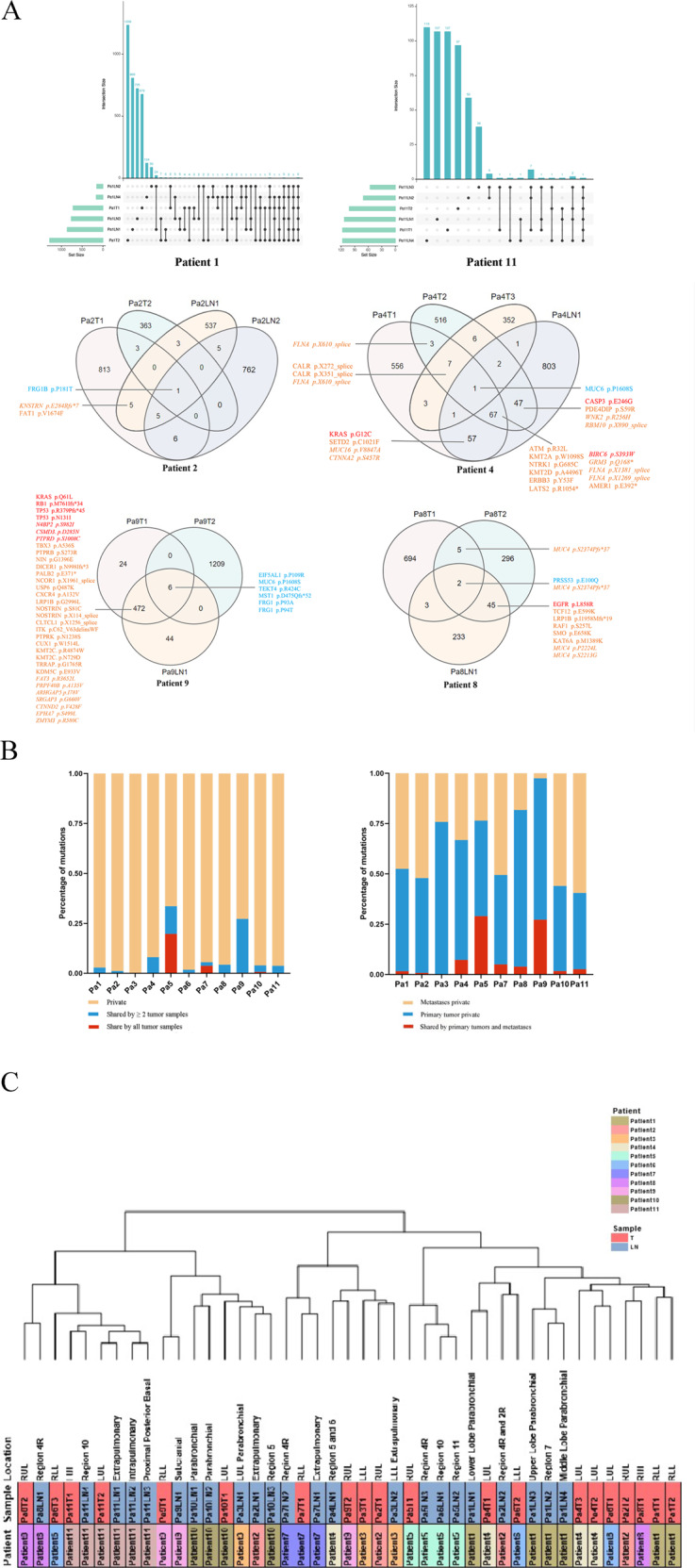
Genomic heterogeneity among different samples. **A** Upset map and Venn diagrams of representative patients showing the distribution of nonsynonymous somatic mutations among different tumors within individuals (Patient 1 and 11: Upset Venn diagrams. Patient 2, 4, 8, and 9: Venn diagrams). The putative pathogenic mutations were marked with different colors and typefaces according to the oncogene list in COSMIC Cancer Gene Census (https://cancer.sanger.ac.uk/census). Orange, Pan-cancer driver gene; Red, NSCLC/lung cancer driver gene; Blue, genes are not pan-cancer driver genes or NSCLC/lung cancer driver gene. Roman type, Tie 1 gene in COSMIC (gene possessing a documented activity relevant to cancer, along with evidence of promoting oncogenic transformation); Italic, Tie 2 gene in COSMIC (genes with strong indications of a role in cancer but with less extensive available evidence). **B** Distributions of mutations. Left: within each individual patient, the percentages of mutations shared by all the tumor samples (Red), mutations shared by two or more tumor samples (Blue), and mutations private to only one tumor sample (Yellow), respectively. For each patient, both the primary lung tumors and the lymph node metastases tumors are identified as tumor samples, regardless of their locations. Right: within each individual patient, the percentages of mutations shared by primary lung tumors and lymph node metastases (Red), mutations private to primary lung tumor samples (Blue), and mutations private to lymph node metastasis samples (Yellow), regardless of the number of tumor samples. **C** The unsupervised clustering of all 11 patients based on non-synonymous mutations. All 11 patients are distinguished with different colors. Red, primary tumor. Blue, lymphatic metastasis tumors.

The Upset map and Venn diagram show the mutation repertoire overlap within representative individuals (Fig. 3A, Supplementary Data 9, Supplementary Fig 2, 3.). For the 9 patients with matched normal samples of our study, the mean number of genes shared by all tumor samples within the individual patient (regardless of the tumor locations) was 3.67 (range 1–10). FRG1B (LOC102724813) mutation was shared by all tumor samples of Patient 1, 2, 6, and 9. Within Patient1, both SETD2 p.X1529_splice and STAG2 (Stromal Antigen 2) p.D1014E were identified by Patient 1T2, Patient 1LN2, and Patient 1LN4. SETD2 is a histone lysine methyltransferase playing a significant role in renal malignancies [28], prostate cancer [24], and NSCLC [29]. STAG2 is proven to be involved with viral infection via STING signaling [30]. CEP192 p.L1701F, a gene involved with PLK1 activity regulation at G2/M Transition, mitotic centrosome maturation, and bipolar assembly [31, 32], was the only mutation common to all 6 tumor samples of Patient 11.

Mutation distribution analysis reveals that MPLC with lymph node metastasis has high intratumor heterogeneity. The genomic complexity of pulmonary and lymph node metastasis tumors is hard to gauge (Fig. 3B, Supplementary Data 10). For all patients, the mutations private to only one tumor sample within a patient account for the most significant proportion (ratio range 66.40–99.72%, median 96.08%; number range 518 to 6721, median 1596), indicating enormous heterogeneity among tumors. For Patient 5 and Patient 7, the percentages of mutations shared by all tumor samples within individual patients are higher than that of other patients (Patient 5, 19.74%, Patient 7, 3.74%), we believe it results from that the two patients lack matched normal samples. Generally, pulmonary tumor samples take larger percentages of private mutations than lymph node metastasis tumor samples (pulmonary, range 37.92–77.86%, mean 55.35%; lymph node metastasis, range 2.51–59.48%, mean 36.71%. *p* = 0.0245), emphasizing the significance of primary tumor management. However, in Patient 2, 7, 10, and 11, lymph node metastasis tumors claim more private mutations than pulmonary tumors, suggesting that lymph node metastasis tumors may be more complex in the genomic background. We visualized the intratumor heterogeneity by global unsupervised clustering analysis (Fig. 3C, Supplementary Data 11). Patient 11 was the only patient whose tumors were closely clustered, except Patient 5 and 7, who lacked paired normal samples. Pulmonary tumors and lymph node metastasis tumors from distinct individuals tended to be clustered together, respectively, implying genomic differences between primary lung tumors and lymph node metastasis sites.

### Clonal architecture and evolutional trajectory

**Fig. 4:**
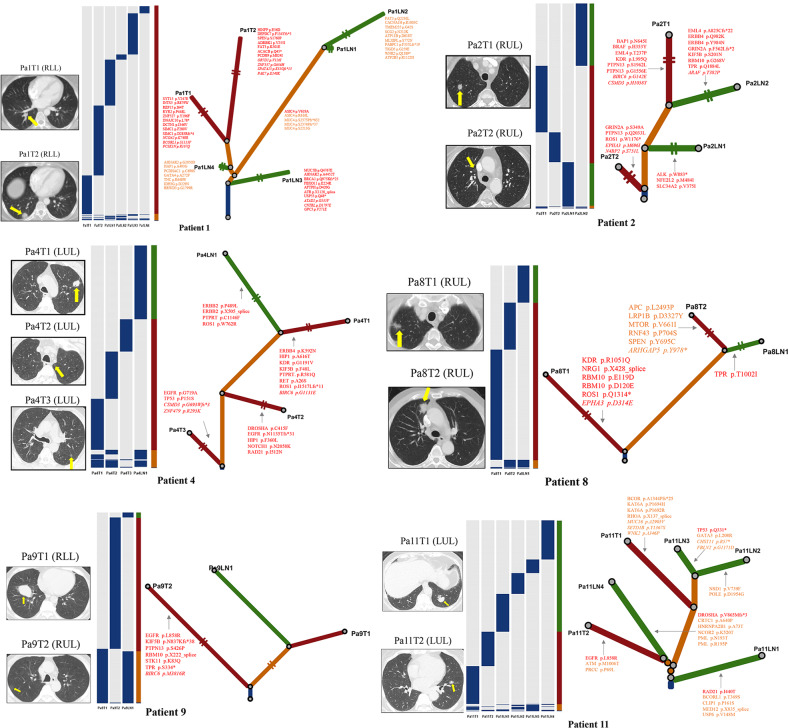
Clonal architecture and evolution of representative patients. Left: CT (computerized tomography) images of each patient, with yellow arrows marking the tumor’s location. Middle: Heatmaps show the distribution of all non-silent mutations; presence (blue), and absence (gray). Colum next to the heatmap shows the distribution of mutations within each individual patient; mutations present in all tumor samples (blue), shared in more than one but not all tumor samples (orange), in only one lung tumor sample (red), and in only one lymph node metastasis tumor sample (green). Right: Phylogenetic trees based on the distribution of all detected mutations. Trunk and branch lengths are proportional to the number of non-silent mutations acquired. Putative driver genes are indicated next to the trunk or with an arrow pointing to the branches where they were detected. Orange, Pan-cancer driver gene; Red, known NSCLC/lung cancer driver gene; Roman type and Italic represent tie 1 gene and tie 2 genes in COSMIC, respectively.

Filtered nonsynonymous variances from each tumor sample were used to construct phylogenetic trees using the parsimony ratchet method [33]. The branch lengths were proportional to the number of nonsilent alterations within individuals (the scales were different among patients for better visual effect), and the driver genes were annotated next to the branches. Individual CT (computerized tomography) images before surgery were provided to validate the multiple pulmonary tumors. Heatmaps were used to show mutation distribution (Fig. 4, Supplementary Data 9, Supplementary Fig 4.). Based on the driver gene list from COSMIC Cancer Gene Census (Sep. 2021, https://cancer.sanger.ac.uk/census), we annotated the driver genes to the branches.

We observed diverse evolutional patterns between primary pulmonary tumors and lymph node metastases. Firstly, in Patient 1, there were 2 lymph node tumors (Pa1LN3 and Pa1LN4) that were earlier in evolution than the two pulmonary tumors. The same phenomenon was found in Patient 11LN1, indicating that lymph node metastasis could be an early event in the pathogenesis of MPLC. Secondly, for Patient 4, 8, and 9, lymph node metastasis lesions were more closely related to the specific individual pulmonary tumor, suggesting these pulmonary tumors were more aggressive in nature. Thirdly, in Patient 2 and 11, lymph node metastases were associated with more than one pulmonary tumor, indicating that multiple pulmonary lesions of MPLC could contribute to metastasis within individuals, for whom radical resection is necessary. Fourthly, we observed that the multiple lymph node metastases tumor samples within the same patient could be very close in evolution. For example, Pa1LN1 and Pa1LN2 in Patient1, Pa5LN1 and Pa5LN2 in Patient 5, Pa10LN1 and Pa10LN2 in Patient 10, and Pa11LN2 and Pa11LN3 in Patient 11. These findings imply the metastasis potentiality of lymph node tumors, and there might be the dominant lesions among multiple lymph node metastases. Moreover, for Patient 3, 5, 7, and 10, their results were consistent with Patient 4, 8, and9, but needed further exploration since each of them had only one valid pulmonary tumor. The three pulmonary tumors of the Patient6 were highly heterogeneous in genetic background, suggesting the intricate nature of MPLC.

Generally, the diverse evolution patterns suggested the complex nature of MPLC with LNM.

#### Immune repertoires of MPLC with LNM

**Fig. 5:**
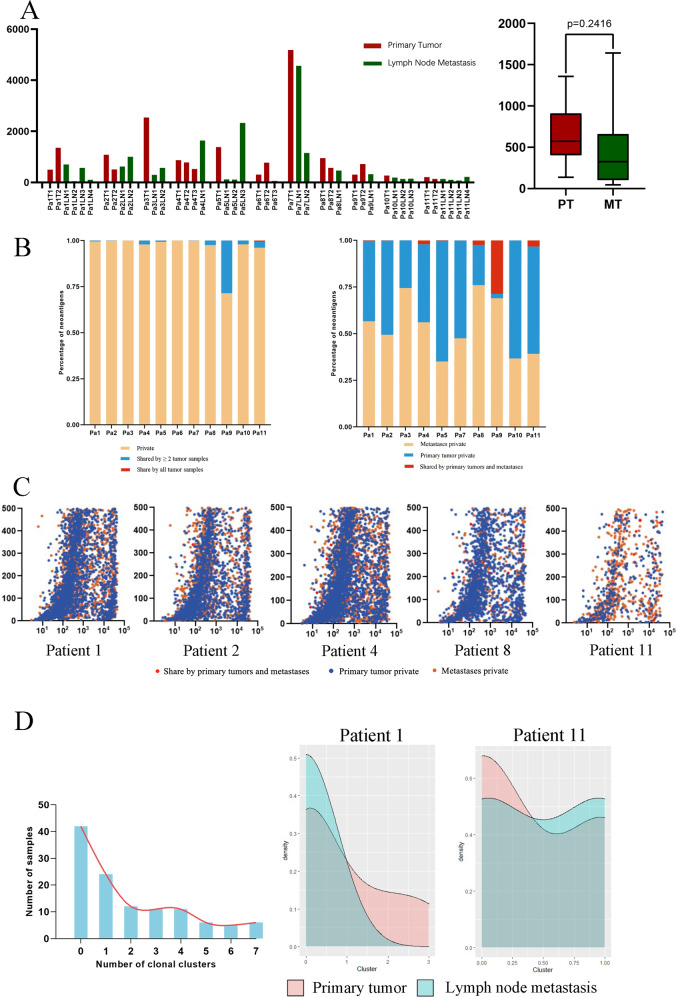
Immunogenicity heterogeneity across and within individuals. **A** Left: The number of all predicted neoantigens in each tumor of 11 patients. Right: The difference in the mean number of potential neoantigens in primary tumors and lymph node metastasis tumors based on Patient 1, 2, 4, 8, 9, and 11 (*p* value = 0.2416). The *X*-axis represents the sample, and the *Y*-axis represents the number of predicted neoantigens of each sample. **B**. Distributions of predicted neoantigens. Left: within each individual patient, the percentages of neoantigens shared by all the tumor samples (Red), shared by two or more tumor samples (Blue), and private to only one tumor sample (Yellow) in each patient, respectively. For each patient, both the primary lung tumors and the lymph node metastases tumors are identified as tumor samples, regardless of their locations. Right: within each individual patient, the percentages of neoantigens shared by primary lung tumors and lymph node metastases (Red), private to primary lung tumor samples (Blue), and private to lymph node metastasis samples (Yellow), regardless of the number of tumor samples. **C** Predictions of neoantigen binding affinity across all 9-11 amino acids peptides generated from nonsynonymous mutations and the matched wild-type peptides using NetMHCpan algorithms. Red, neoantigens shared by both primary and lymph node metastasis tumors within one individual patient; Blue, neoantigens private to primary lung tumor samples of one individual patient; Orange, neoantigens private to lymph node metastasis tumor samples of one individual patient. **D** Histograms and density plots of SciClone inferred clonal clusters. Left: Clonal clusters of all 11 patients. Right: Clonal clusters density plots of Patient 1 and 11.

Currently, the major management for MPLC is surgical resection, and the clues of chemotherapy or immunotherapy are rare. To offer new insights into the immunotherapy administration in MPLC, we performed neoantigen number prediction, neoantigen binding affinity prediction, and clone cluster calculation in Fig. 5. Overall, multiple tumors in MPLC showed extremely high heterogeneity in the immune background, which is more complicated than the mutational spectrum. Both primary pulmonary tumors and lymph node metastasis tumors could have diverse clone clusters. In Fig. 5A (Supplementary Data 12), we showed the predicted neoantigen number of each sample. For key patients (Patient 1, 2, 4, 8, 9, and 11), the mean predicted neoantigen number of primary lung cancers (mean 646.5, range 59–5186) was higher than that of lymph node metastases (mean 300, range 45–4562). Still, this discrepancy was not significant statistically (*p* = 0.2416). For all 11 patients, neoantigens private to only one tumor sample possessed absolute advantage (range 71.40–100%, mean 99.36%). In Patient 3 and 6, all their predicted neoantigens were private to one tumor (Fig. 5B Left, Supplementary Data 13). Neoantigens of primary lung tumors (range 35.07–76.02%, mean 52.72%) hold similar proportions to that of lymph node metastases (range 2.38–64.70%, mean 46.80%), while the two parts shared few neoantigens (range 0–28.60%, mean 0.27%), suggesting the two parts might response to immunotherapy in different manners (Fig. 5B Right, Supplementary Data 13). Binding affinity analysis shows that primary lung tumors and lymph node metastases harbor distinct neoantigens repertoires (Fig. 5C, Supplementary Data 14), implying heterogeneous tumor microenvironments and potentially various reactions to immune checkpoint blockers. Most sequenced samples were oligoclonal, and the clonal structures of the primary tumor and metastases were in diverse patterns (Fig. 5D, Supplementary Data 15).

## Discussion

In our study, the mutation landscape of MPLC with LNM is quite different from that of east Asian LUAD patients [34] in our study, suggesting there might be unique features of this disease and the necessity of further research. Our study’s most frequently mutated oncogenes were MUC16, FLNA, MUC4, FAT3, SETD2, and CALR, all of which were pan-cancer driver genes, and no highly mutated lung cancer driver gene ranked in the top 20 mutations, such as EGFR, KRAS, and TP53. This deviation might result from the insufficient sequencing depth of a few samples in our study. Still, we have reason to infer that such inconsistencies indicate that MPLC with LNM may have a unique mutation spectrum, which needs further validation in larger cohorts. SETD2 (SET Domain Containing 2) mutated in all 6 tumors of the Patient 1 (five of them were splice-site mutations), suggesting its potentiality to be a driving force of lymph node metastasis in MPLC. It is a histone lysine methyltransferase, closely involved with cancer behavior [35, 36], and recurrently mutated in several cancer types [24, 37]. Moreover, SETD2 mutated in one pulmonary tumor of an MPLC with an LNM patient in a previous study [13], which was partly consistent with our results. TTN (Titin), the gene with the highest alteration rate in our cohort, codes a sarcomere protein which is a significant player in cardiomyocyte stiffness and cardiac train sensing [38]. In lung cancer, several recent studies reported that TTN mutations enhanced anti-tumor immunity and were associated with favorable responses to ICI administration [39–41]. Therefore, we believe that MPLC and MPLC with LNM might be ideal substrates for ICI.

Our study imposed further deliberation on the current MPLC diagnosis standard. In the clinical practice of multifocal lung cancer, precise differentiation between MPLC and IPM has been a dilemma for decades [42], rendering treatment selection challenging [9–11]. Many previous studies have paid attention to this field [6, 11–13, 43–48]. They believed that at most 1 mutation could be shared by any two pulmonary tumors of MPLC. And the patients whose pulmonary tumors share two or more than two (≥2) mutations should be defined as IPM [11]. However, in our study, the shared mutations by any two pulmonary tumor samples within an individual patient ranged from 1 to 78 (median: 20, Patient 3, 5, 7, and 10 were excluded for they only had one valid lung tumor, thus could not perform this analysis), inconsistent with previous studies, and very few overlap genes were oncogenes. This inconsistency could result from the sequencing strategies used by previous studies, most of them chose targeted sequencing, which only detected cancer-related genes [11, 49, 50], while WES/WGS was applied to only a few patients [12, 43]. Our research indicates that multiple pulmonary tumors of MPLC could be more like each other in the genomic profile than we know before, and it might be arbitrary to distinguish MPLC and IPM under the current “1 mutation” criteria. Moreover, consistent with Qu et al.’s work [51], our study suggests that the absence of LNM may not be necessary for diagnosing MPLC with similar tumor pathology. MPLC with LNM should be carefully diagnosed to guarantee that the patients do not miss the best treatment opportunity.

We offered novel clues for identifying the “dominant” lung tumors in MPLC and challenged the current recognition of MPLC. At present, the clonal evolution relationships among different tumor lesions of MPLC with lymph node metastasis are unclear but of great significance, since we may catch the driving force dictating the metastasis progression and identify the tumor site that has the most potential for metastasis so that doctors can react in advance, and get a thorough understanding of the biological behavior of MPLC. However, these works are hampered by the enormous genetic heterogeneity of MPLC. While in MPLC with LNM, the genetic trajectory between primary pulmonary tumor and lymph node metastasis is an ideal material to illustrate this dilemma. In 2016, Liu et al. [12] firstly sequenced one MPLC with an LNM patient who was adenocarcinoma. In 2020, Higuchi et al. [13] performed targeted sequencing on 5 MPLC with LNM patients whose pulmonary tumors were all LUSC in histology. These two studies indicated that lymph node metastasis was genetically related to one specific lung lesion, suggesting metastasis dominance. In our research, we drew phylogenetic trees for each patient and inferred the evolutional process of multiple tumors based on the theory of clonal heterogeneity and tumor evolution [52]. We found that the lymph node metastases could originate from both one and multiple pulmonary tumors within the individual, indicating that the “dominant” tumors could be more than one. In addition, our results challenge the current understanding that MPLC is early in staging since some lymph node metastases are evolutionally earlier than pulmonary tumors.

For clinical doctors, we provided new choices for MPLC management by advancing dominant tumor identification. Currently, the most prevalent treatment for MPLC is surgical resection [53, 54]. However, many therapies are feasible in MPLC, including photodynamic therapy [55], stereotactic ablative radiotherapy [56], and a combination of local radiofrequency ablation and melatonin [57]. When the multiple tumors of MPLC are in different lobes or lungs, multiple operations become necessary, which is riskier than one-time resection. However, many MPLC patients cannot endure highly radical resections because of surgical contraindications. Therefore, if we can pre-locate and remove the dominant tumor, more patients will benefit from surgery more safely. In addition, recurrence is widespread for multiple lung cancer in clinical practice, the underlying reasons for which are unclear. At the same time, dominant tumor resection can be a critical indicator for recurrence prevention.

We offered novel insights for the immunotherapy administration in MPLC and MPLC with LNM. In the recent decade, immune checkpoint inhibitors achieved considerable success in multiple malignancies [58], including NSCLC [59] (Non-Small Cell Lung Cancer). However, MPLC and MPLC with LNM are seldom involved. In Fig. 5, we observed that the multiple tumors within individuals were highly heterogeneous in neoantigen, while disparities exist between primary tumors and metastases. Our findings shed light on the application of ICIs (immune checkpoint inhibitors) on MPLC and MPLC with LNM and suggested that different strategies should be applied to primary and metastases samples. There are mainly three obstacles in unveiling the nature of MPLC and MPLC with LNM: Firstly, the lack of a golden standard or generally accepted definitions of MPLC hindered the integration of data generated by different researchers who might adopt other criteria. Secondly, it is not easy to collect MPLC samples in surgery since some tumor nodules are too small to resect, and the multiple nodules might locate on different sides of the lungs. In contrast, only one side of the lung will be touched in one surgery. Thirdly, WES or WGS (whole-genome sequencing) still cannot be afforded by most scientific institutes, so most researchers used target sequencing, which could not give a profound coverage of the genomic profile of MPLC. In our study, the incidence of MPLC with LNM was 2.54% (37/1458), and we estimate that the incidence is higher in the real world. Therefore, it is significant to study this malignancy regardless of several obstacles.

Several limitations existed in our study. Firstly, due to the limited number of patients (only 11 cases were included) and the lack of large-scale validation cohorts in this study, most conclusions we reached should be interpreted with caution. Secondly, a few samples failed in the WES quality control, causing imperfection in some patients. Thirdly, the sequencing depth of many samples is insufficient for producing highly valid results, many key mutations were missing. Fourthly, we only performed WES on the patients in our study. Therefore, we missed the data in the non-coding area of the genome, which contains significant biological information. RNA-seq and profiling of other omics, such as methylation, were not performed in our study, limiting our perception of this disease.

In conclusion, by characterizing the molecular features of MPLC with LNM, we identified potential driver genes of this disease. We revealed various evolution patterns among primary pulmonary and lymph node metastasis tumors. We found that lymph node metastasis might be an early event in the etiology of MPLC. Our findings push the boundaries of MPLC and MPLC with LNM by providing new information for dominant tumor identification. Further solid evidence of MPLC and MPLC with LNM is warranted.

## Supplementary information

Supplementary Data 1

Supplementary Data 2

Supplementary Data 3

Supplementary Data 4

Supplementary Data 5

Supplementary Data 6

Supplementary Data 7

Supplementary Data 8

Supplementary Data 9

Supplementary Data 10

Supplementary Data 11

Supplementary Data 12

Supplementary Data 13

Supplementary Data 14

Supplementary Data 15

Supplementary Figure 1

Supplementary Figure 2

Supplementary Figure 3

Supplementary Figure 4

Supplementary Figure 5

Supplementary Figure 6

Supplementary Materials Legend (For Supplementary Figures)

## Data Availability

We deposited our data in the Genome Sequence Archive-Human in BIG Data Center, Beijing Institute of Genomics (BIG), under accession number HRA001717 (https://bigd.big.ac.cn/gsa-human/browse/HRA001717).
