# Supplementary figures and images for: Genetic trajectory and clonal evolution of multiple primary lung cancer with lymph node metastasis
Source: Cancer Gene Ther. 2023 Jan 19;30(3):507–20. doi: 10.1038/s41417-022-00572-0 (PMC10014582; doi:10.1038/s41417-022-00572-0)

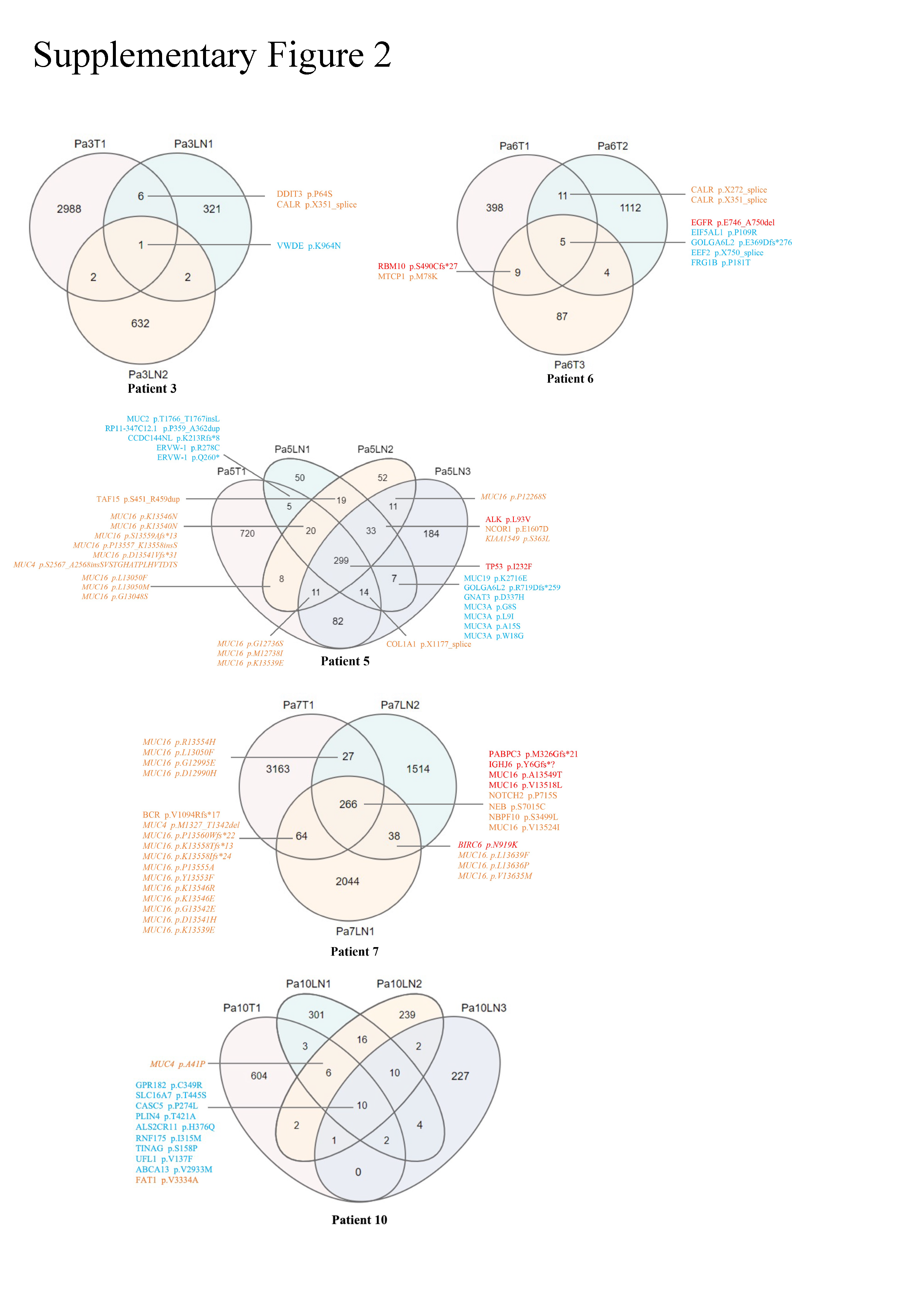

Supplement: Supplementary file 17 — Supplementary Figure 2 [file 41417_2022_572_MOESM17_ESM.tif]

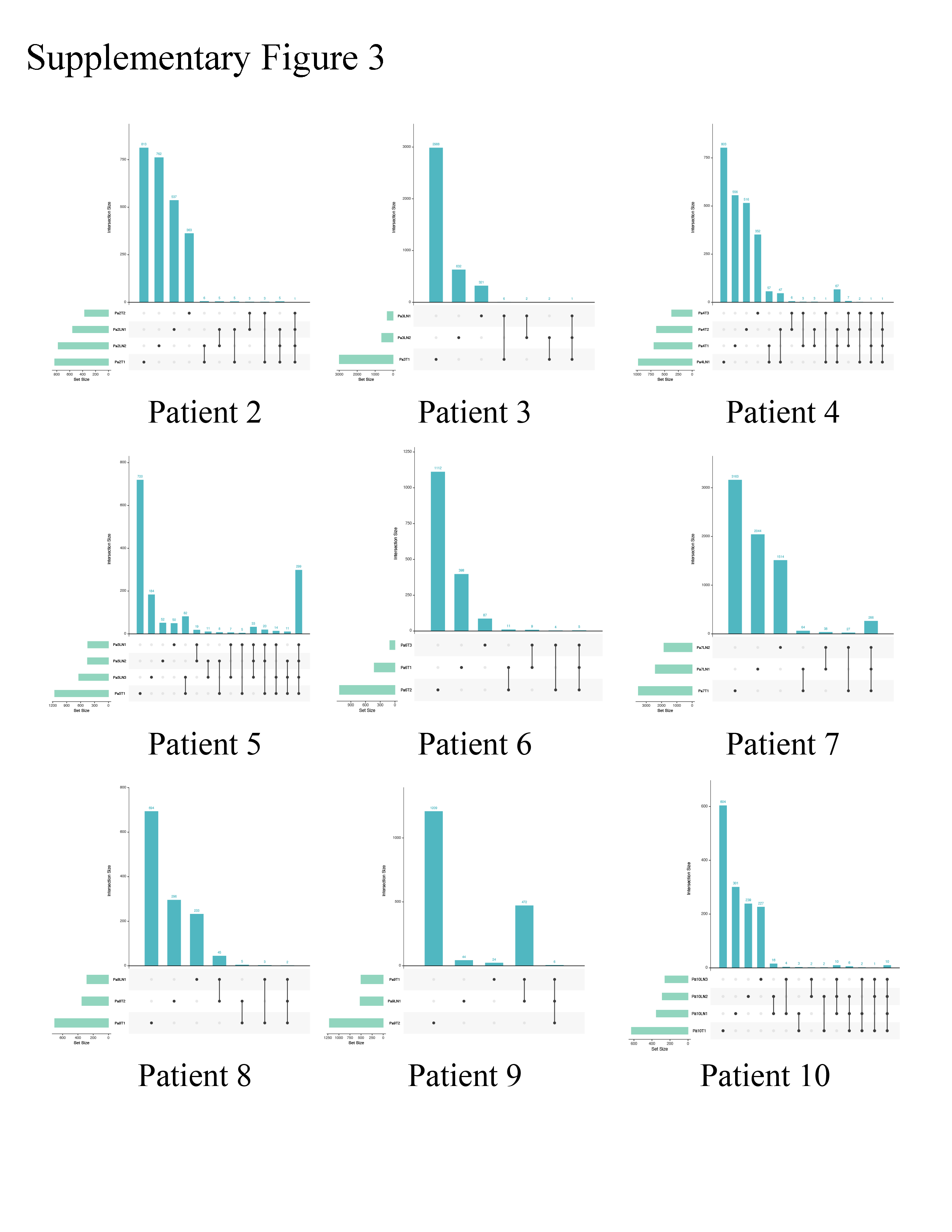

Supplement: Supplementary file 18 — Supplementary Figure 3 [file 41417_2022_572_MOESM18_ESM.tif]

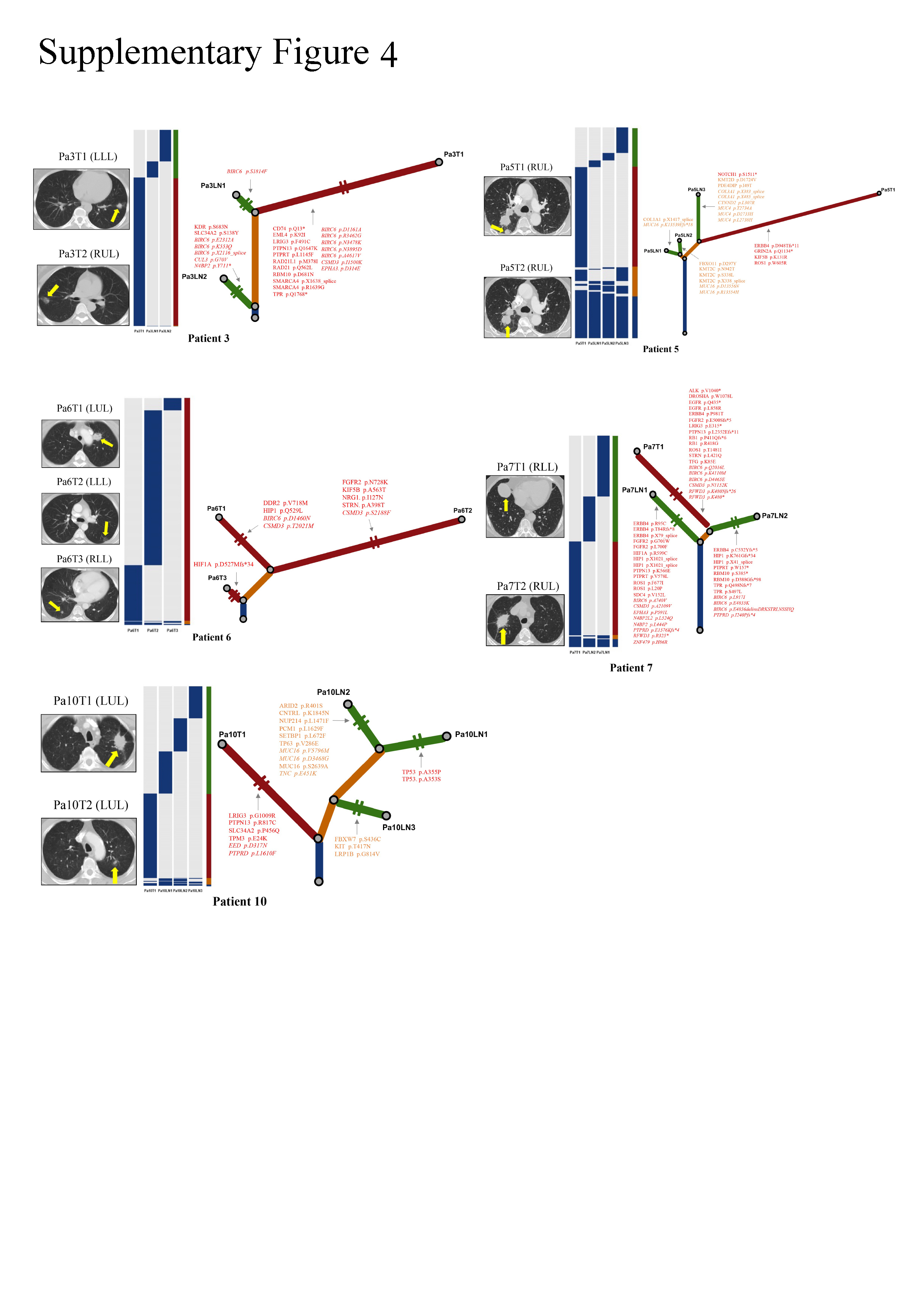

Supplement: Supplementary file 19 — Supplementary Figure 4 [file 41417_2022_572_MOESM19_ESM.tif]

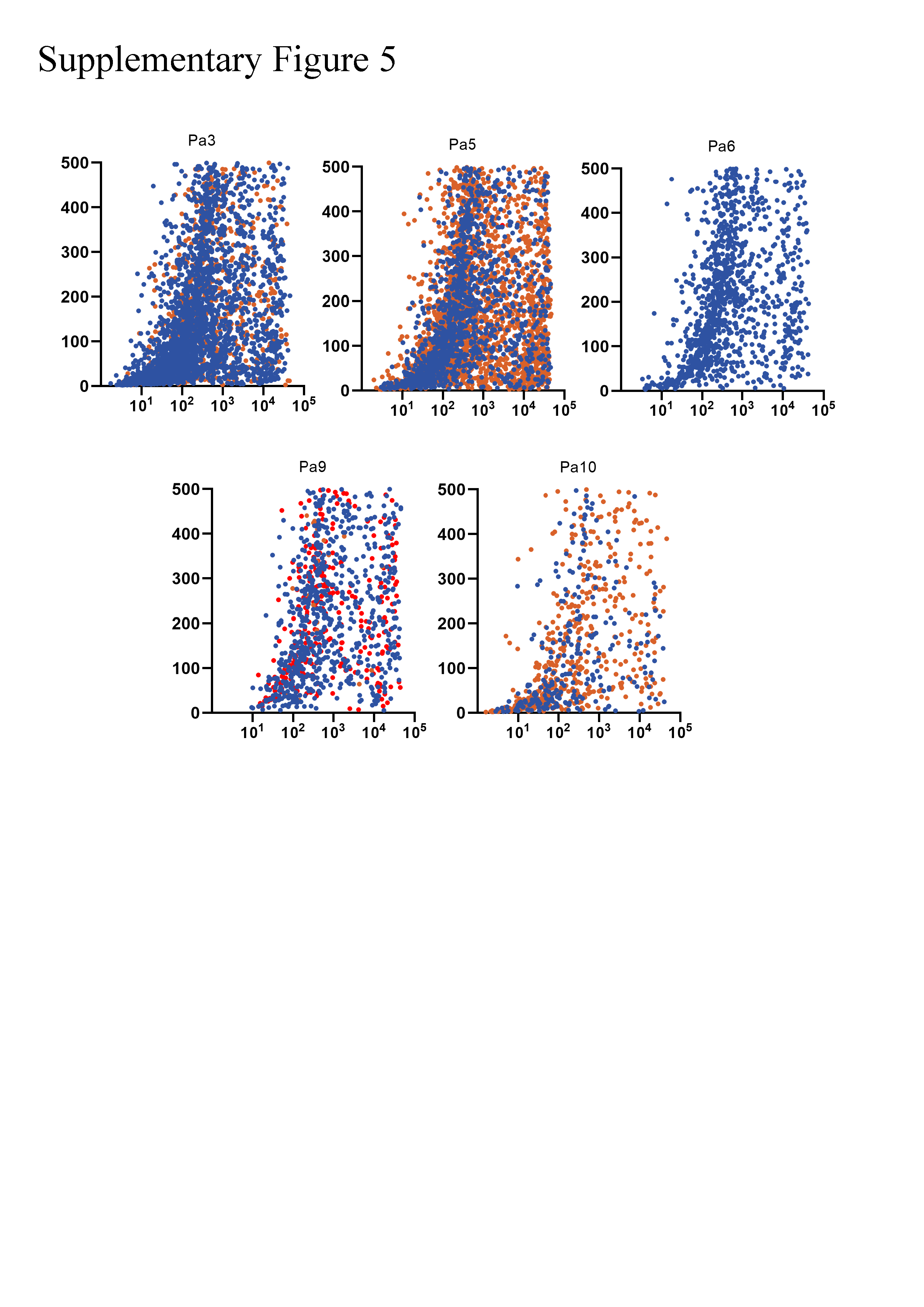

Supplement: Supplementary file 20 — Supplementary Figure 5 [file 41417_2022_572_MOESM20_ESM.tif]

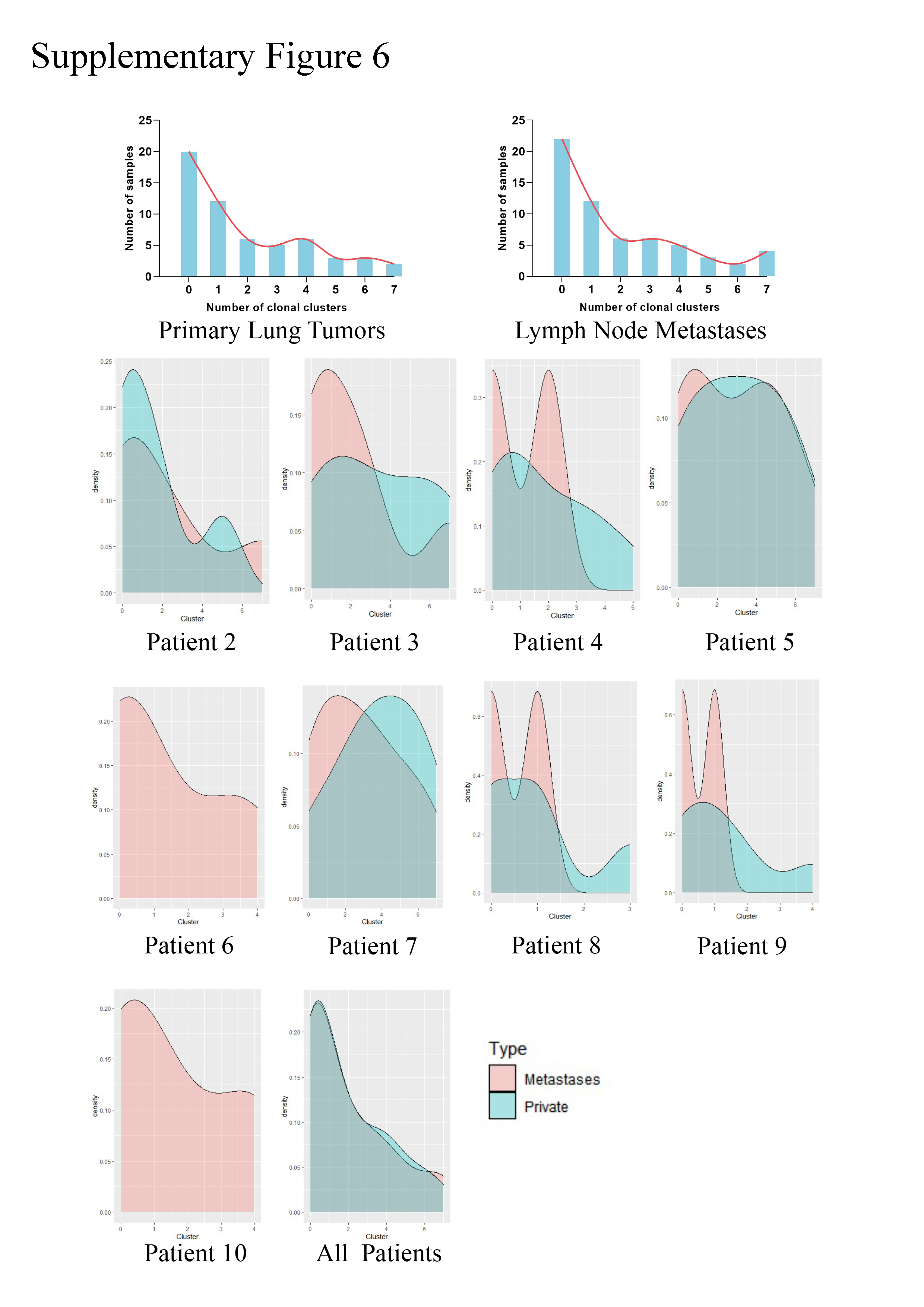

Supplement: Supplementary file 21 — Supplementary Figure 6 [file 41417_2022_572_MOESM21_ESM.tif]
