# Supplementary material for: Genetic trajectory and clonal evolution of multiple primary lung cancer with lymph node metastasis
Source: Cancer Gene Ther. 2023 Jan 19;30(3):507–20. doi: 10.1038/s41417-022-00572-0 (PMC10014582; doi:10.1038/s41417-022-00572-0)
Supplement: Supplementary file 22 — Supplementary Materials Legend (For Supplementary Figures) [file 41417_2022_572_MOESM22_ESM.docx]

**Supplementary Figure Legend**

**Supplementary Figure1.** Representative HE staining image of all 11 patients in our study. (Magnification: 100X. Scale bars: 200μm).

**Supplementary Figure2.** Venn diagrams of Patient 3, 5, 6, 7, and 10, showing the distribution of nonsynonymous somatic mutations among different tumors within individuals. The putative pathogenic mutations were marked with different colors and typefaces according to the oncogene list in COSMIC Cancer Gene Census (https://cancer.sanger.ac.uk/census). Orange, Pan-cancer driver gene; Red, N SCLC/lung cancer driver gene; Blue, genes are not pan-cancer driver genes or NSCLC/lung cancer driver gene. Roman type, Tie 1 gene in COSMIC (gene possessing a documented activity relevant to cancer, along with evidence of promoting oncogenic transformation); Italic, Tie 2 gene in COSMIC (genes with strong indications of a role in cancer but with less extensive available evidence).

**Supplementary Figure3.** Upset maps of Patient 2, 3, 4, 5, 6, 7, 8, 9, and 10, showing the distribution of nonsynonymous somatic mutations among different tumors within individuals.

**Supplementary Figure4.** Clonal architecture and evolution of Patient 3, 5, 6, 7, and 10. **Left**: CT (computerized tomography) images of each patient, with yellow arrows marking the tumor's location. **Middle**: Heatmaps show the distribution of all non-silent mutations; presence (blue), and absence (gray). Colum next to the heatmap shows the distribution of mutations within each individual patient; mutations present in all tumor samples (blue), shared in more than one but not all tumor samples (orange), in only one lung tumor sample (red), and in only one lymph node metastasis tumor sample (green). **Right**: Phylogenetic trees based on the distribution of all detected mutations. Trunk and branch lengths are proportional to the number of non-silent mutations acquired. Putative driver genes are indicated next to the trunk or with an arrow pointing to the branches where they were detected. Orange, Pan-cancer driver gene; Red, known NSCLC/lung cancer driver gene; Roman type and Italic represent tie 1 gene and tie 2 genes in COSMIC, respectively.

**Supplementary Figure5.** Predictions of neoantigen binding affinity across all 9-11 amino acids peptides generated from nonsynonymous mutations and the matched wild-type peptides using NetMHCpan algorithms for Patient 3, 5, 6, 9, and 10. Red, neoantigens shared by both primary and lymph node metastasis tumors within one individual patient; Blue, neoantigens private to primary lung tumor samples of one individual patient; Orange, neoantigens private to lymph node metastasis tumor samples of one individual patient.

**Supplementary Figure6.** Histograms and density plots of SciClone inferred clonal clusters. Upper: Clonal clusters of primary lung tumors and lymph node metastases, respectively. Lower, Clonal clusters density plots of Patient 2, 3, 4, 5, 6, 7, 8, 9, 10, and all 11 patients.
